# Supplementary material for: Rab7a is an enhancer of TPC2 activity regulating melanoma progression through modulation of the GSK3β/β-Catenin/MITF-axis
Source: Nat Commun. 2024 Nov 19;15:10008. doi: 10.1038/s41467-024-54324-9 (PMC11576762; doi:10.1038/s41467-024-54324-9)
Supplement: Supplementary file 1 — Supplementary Information [file 41467_2024_54324_MOESM1_ESM.pdf]

Supplementary information

Rab7a is an enhancer of TPC2 activity regulating melanoma progression through modulation of the GSK3β/β-Catenin/MITF-axis

Carla Abrahamian[1,2#], Rachel Tang[1#], Rebecca Deutsch[1#], Lina Ouologuem[3#], Eva-Maria Weiden[1], Veronika Kudrina[1], Julia Blenninger[3], Julia Rilling[3], Colin Feldmann[4], Solveig Kuss[5], Youli Stepanov[6], Anna Scotto Rosato[1], Guadalupe T. Calvo[7], Maria S. Soengas[7], Doris Mayr[5], Thomas Fröhlich[6], Thomas Gudermann[1], Martin Biel[3], Christian Wahl-Schott[4], Cheng-Chang Chen[8,9], Karin Bartel[3]*, Christian Grimm[1,10]*

[1]Walther Straub Institute of Pharmacology and Toxicology, Faculty of Medicine, Ludwig-Maximilians-University, Munich, Germany.
[2]Department of Cardiology, German Heart Centre Munich, Technical University of Munich, Germany.
[3]Department of Pharmacy, Ludwig-Maximilians-University, Munich, Germany.
[4]Institute of Cardiovascular Physiology and Pathophysiology, Faculty of Medicine, Ludwig-Maximilians-University, Munich, Germany.
[5]Institute of Pathology, Faculty of Medicine, Ludwig-Maximilians-University, Munich, Germany.
[6]Laboratory for Functional Genome Analysis LAFUGA, Gene Center, Ludwig-Maximilians-University, Munich, Germany.
[7]Melanoma Laboratory, Molecular Pathology Programme, Centro Nacional de Investigaciones Oncológicas (Spanish National Cancer Research Centre), Madrid, Spain.
[8]Department of Clinical Laboratory Sciences and Medical Biotechnology, College of Medicine, National Taiwan University, Taipei, Taiwan.
[9]Department of Laboratory Medicine, National Taiwan University Hospital, Taipei, Taiwan.
[10]Immunology, Infection and Pandemic Research IIP, Fraunhofer Institute for Translational Medicine and Pharmacology ITMP, Munich/Frankfurt, Germany

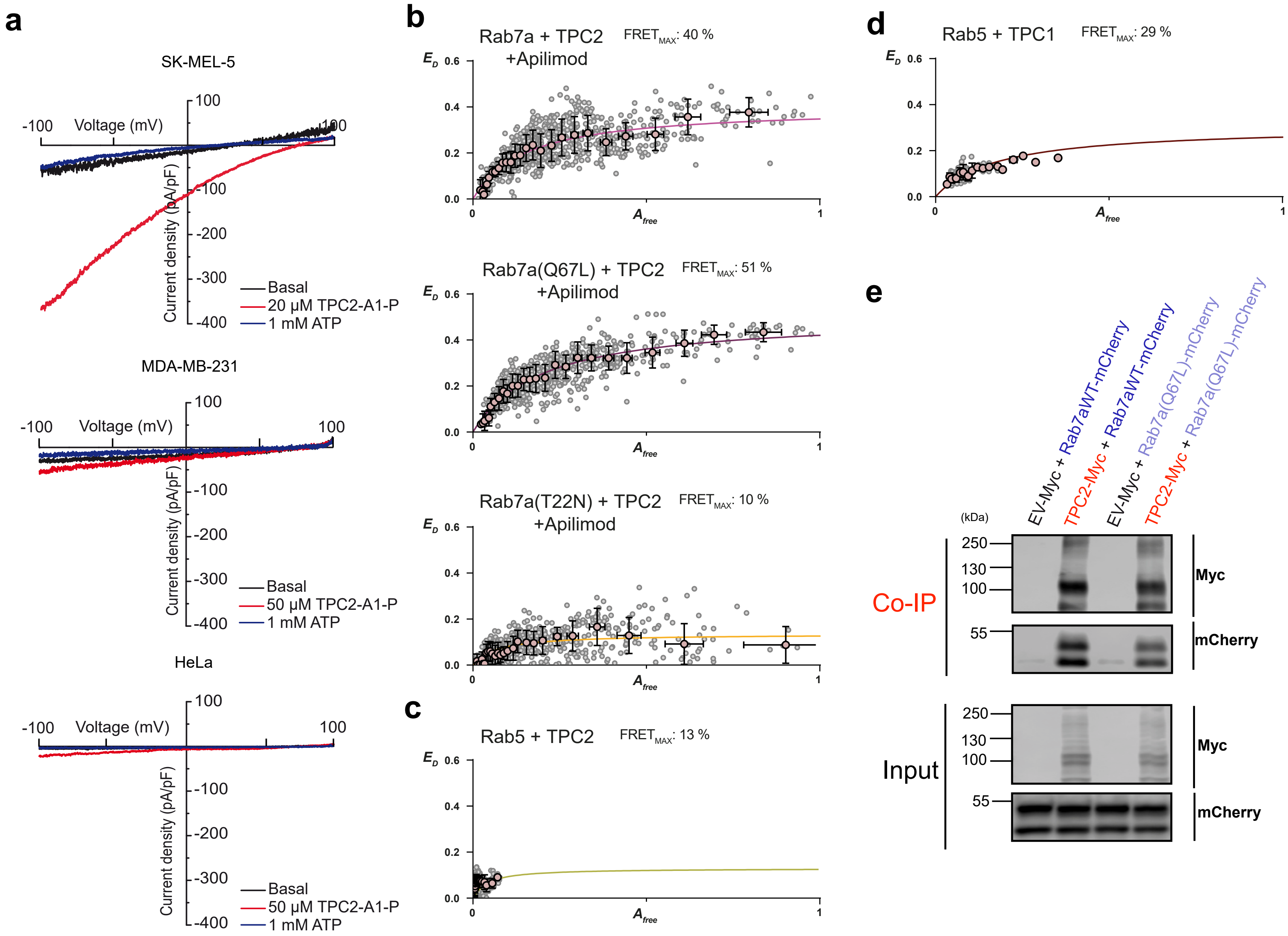
a
SK-MEL-5
Voltage (mV)
Current density (pA/pF)
Basal
20 µM TPC2-A1-P
1 mM ATP
MDA-MB-231
50 µM TPC2-A1-P
HeLa
b
Rab7a + TPC2 +Apilimod
FRETMAX: 40 %
Rab7a(Q67L) + TPC2 +Apilimod
FRETMAX: 51 %
Rab7a(T22N) + TPC2 +Apilimod
FRETMAX: 10 %
c
Rab5 + TPC2
FRETMAX: 13 %
d
Rab5 + TPC1
FRETMAX: 29 %
ED
Afree
e
EV-Myc + Rab7aWT-mCherry
TPC2-Myc + Rab7aWT-mCherry
EV-Myc + Rab7a(Q67L)-mCherry
TPC2-Myc + Rab7a(Q67L)-mCherry
(kDa)
Co-IP
Input
Myc
mCherry

Figure S1. Endolysosomal patch-clamp, FRET and co-immunoprecipitation experiments. (**a**) Endolysosomal patch-clamp experiments showing effect of the TPC2 agonist TPC2-A1-P and the TPC2 blocker ATP on different cancer cell lines. (**b-d**) FRET experiments showing FRET efficiencies in HEK293 cells expressing $hTPC2^{WT}$ + $hRab7^{WT}$ (n = 767), $hTPC2^{WT}$ + $hRab7^{Q67L}$ (n = 676), or $hTPC2^{WT}$ + $hRab7^{T22N}$ (n = 588) in presence of apilimod (a). Shown in c and d are the controls $Rab5^{WT}$ + TPC2 ^{WT} (negative) (n = 536) and $Rab5^{WT}$ + $TPC1^{WT}$ (positive) (n = 87) (n represents biological replicates, error bars are SEM). (**e**) Representative co-immunoprecipitation experiment indicating an interaction between both $hRab7a^{WT}$-mCherry and $hTPC2^{WT}$-YFP and $hRab7a^{Q67L}$-mCherry with $hTPC2^{WT}$-YFP[1].

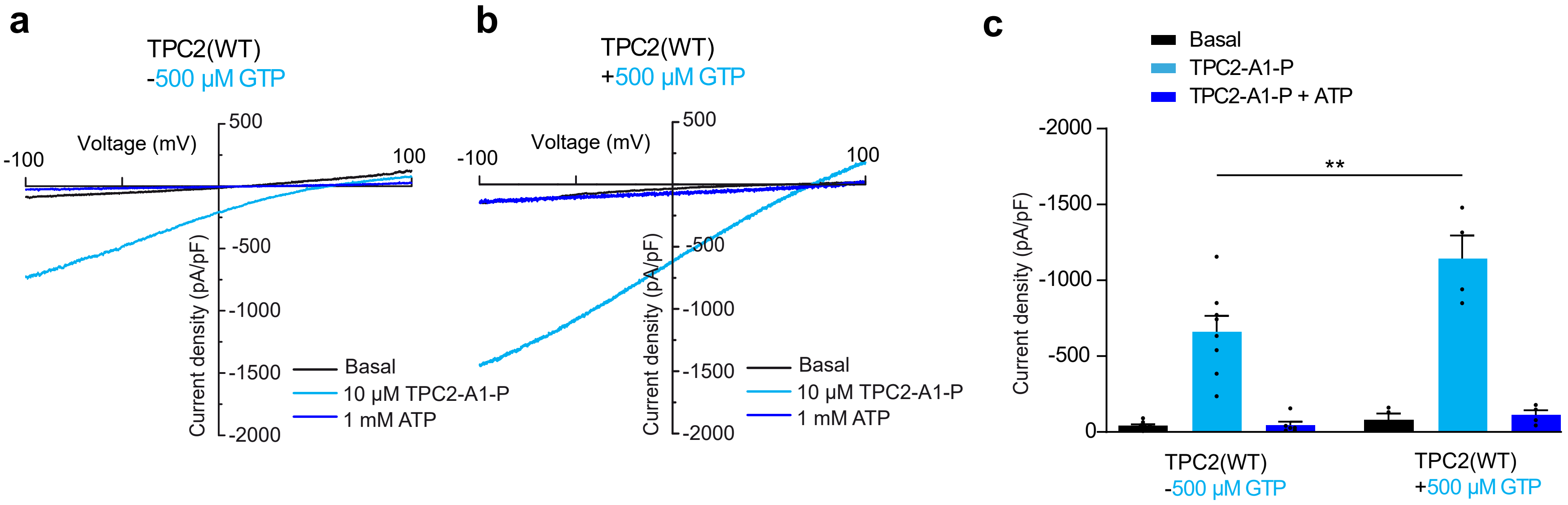
a
TPC2(WT)
-500 μM GTP
Voltage (mV)
-100
100
500
-500
-1000
-1500
-2000
Current density (pA/pF)
Basal
10 μM TPC2-A1-P
1 mM ATP
b
TPC2(WT)
+500 μM GTP
Voltage (mV)
-100
100
500
-500
-1000
-1500
-2000
Current density (pA/pF)
Basal
10 μM TPC2-A1-P
1 mM ATP
c
Basal
TPC2-A1-P
TPC2-A1-P + ATP
-2000
-1500
-1000
-500
0
Current density (pA/pF)
**
TPC2(WT)
-500 μM GTP
TPC2(WT)
+500 μM GTP

Figure S2. Effect of GTP preincubation on TPC2 activity. (**a-b**) Effect of GTP and TPC2-A1-P (10 µM) on apilimod-treated, enlarged endolysosomal vesicles expressing hTPC2. Shown are representative current density-voltage relationships from -100 to +100 mV with basal currents in black, TPC2-A1-P activated currents in the absence or after preincubation (5 min) with GTP in light blue and ATP (1 mM) blocked currents in dark blue. (**c**) Statistical summary of data comprising average current densities (mean ± SEM) at -100 mV measured in endolysosomal patch-clamp experiments as shown in a-b. Each dot on the bar graph represents a single current density value measured from one endolysosome (n = 4-8 biological replicates). Data were tested for statistical significance with one-way ANOVA test followed by Tukey's post-test, $^{**}p < 0.01$.

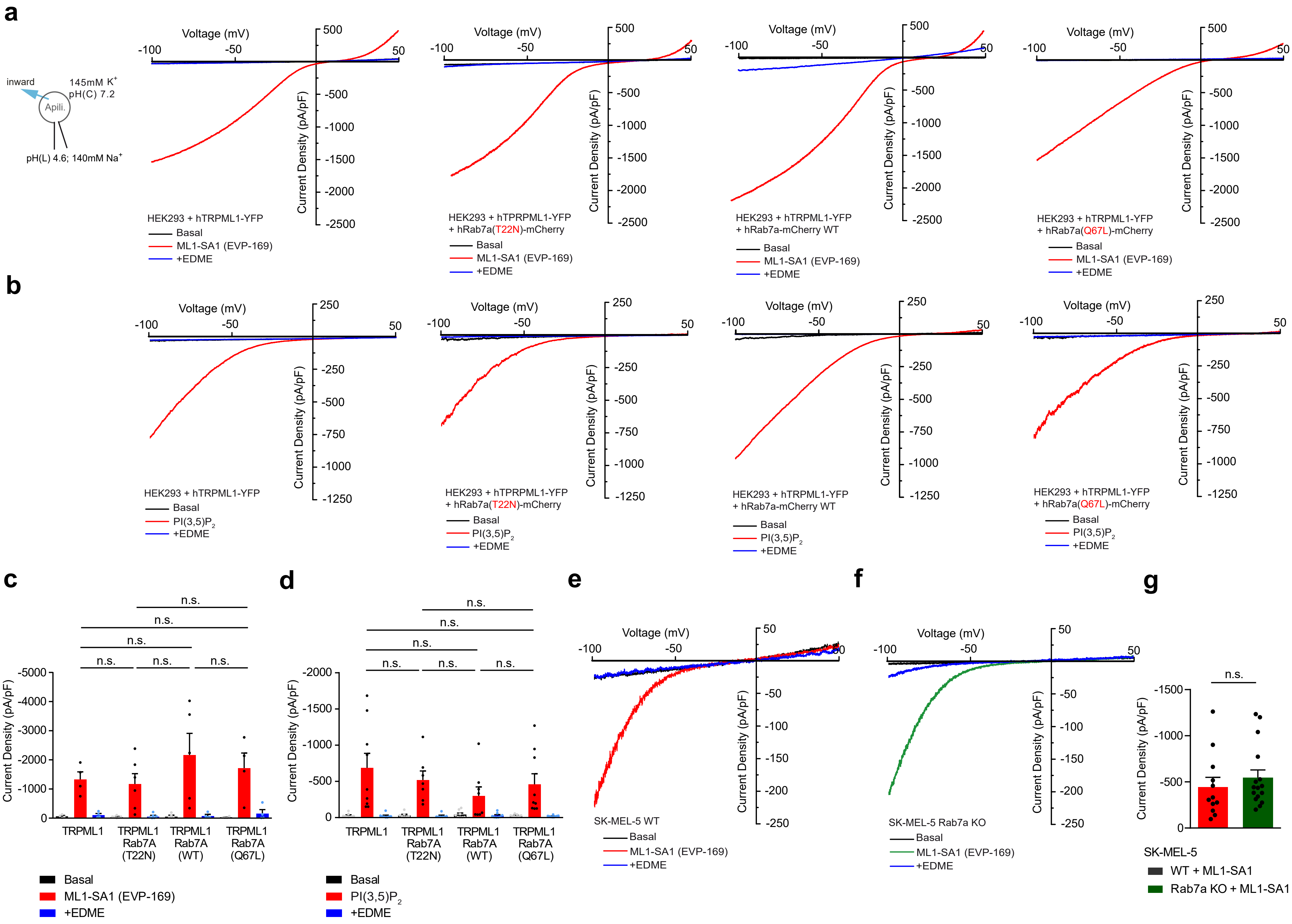

a
inward
145mM K+
pH(C) 7.2
Apili.
pH(L) 4.6; 140mM Na+
Voltage (mV)
Current Density (pA/pF)
-100
-50
50
500
-500
-1000
-1500
-2000
-2500
HEK293 + hTRPML1-YFP
Basal
ML1-SA1 (EVP-169)
+EDME
HEK293 + hTPRPML1-YFP
+ hRab7a(T22N)-mCherry
HEK293 + hTRPML1-YFP
+ hRab7a-mCherry WT
HEK293 + hTRPML1-YFP
+ hRab7a(Q67L)-mCherry
b
250
-250
-500
-750
-1000
-1250
PI(3,5)P2
c
n.s.
-5000
-4000
-3000
-2000
-1000
0
TRPML1
TRPML1 Rab7A (T22N)
TRPML1 Rab7A (WT)
TRPML1 Rab7A (Q67L)
d
-2000
-1500
-1000
-500
e
-50
-100
-150
-200
-250
SK-MEL-5 WT
f
SK-MEL-5 Rab7a KO
g
-1500
SK-MEL-5
WT + ML1-SA1
Rab7a KO + ML1-SA1

Figure S3. Effect of Rab7a on TRPML1 activity. (**a**) Effect of lipophilic small molecule agonist of TRPML1 ML1-SA1 in endolysosomal vesicles coexpressing human TRPML1 and Rab7a or mutant variants of Rab7a. Shown are representative current density-voltage relationships from -100 to +100 mV with basal currents in black, 10 µM ML1-SA1 activated currents in red and EDME (10 µM) blocked currents in blue, measured from apilimod-treated, enlarged endolysosomal vesicles expressing either $hTRPML1^{WT}$, $hTRPML1^{WT}$ + $hRAB7a^{WT}$, $hTRPML1^{WT}$ + $hRAB7a^{Q67L}$ (constitutively active Rab7a) or $hTRPML1^{WT}$ + $hRAB7a^{T22N}$ (dominant negative Rab7a). (**b**) Analogous experiments for $PI(3,5)P_2$ (1 µM). (**c** and **d**) Statistical summary of data comprising average current densities (mean ± SEM) at -100 mV measured in endolysosomal patch-clamp experiments as shown in (a) and (b). Each dot on the bar graph represents a single value current density measured from one endolysosome (for (a) n = 4-6 and for (b) n = 7-9 biological replicates). Data were tested for statistical significance with a one-way ANOVA test followed by Tukey's post-test. (**e** and **f**) Representative current density-voltage relationships from -100 to +100 mV showing basal, ML1-SA1 activated and EDME (10 µM) blocked currents, measured from apilimod-treated, enlarged endolysosomal vesicles, in SK-MEL-5 WT cells (e) and Rab7a KO (f). (**g**) Statistical summary of data comprising average current densities at -100 mV measured in endolysosomal patch-clamp experiments as shown in (e) and (f) elicited with 10 µM ML1-SA1 (WT and Rab7a KO SK-MEL-5 lines, respectively). Each dot on the bar graph represents a single value current density measured from one endolysosome (n = 12-16 biological replicates). Data were tested for statistical significance using a two-tailed unpaired t-test.

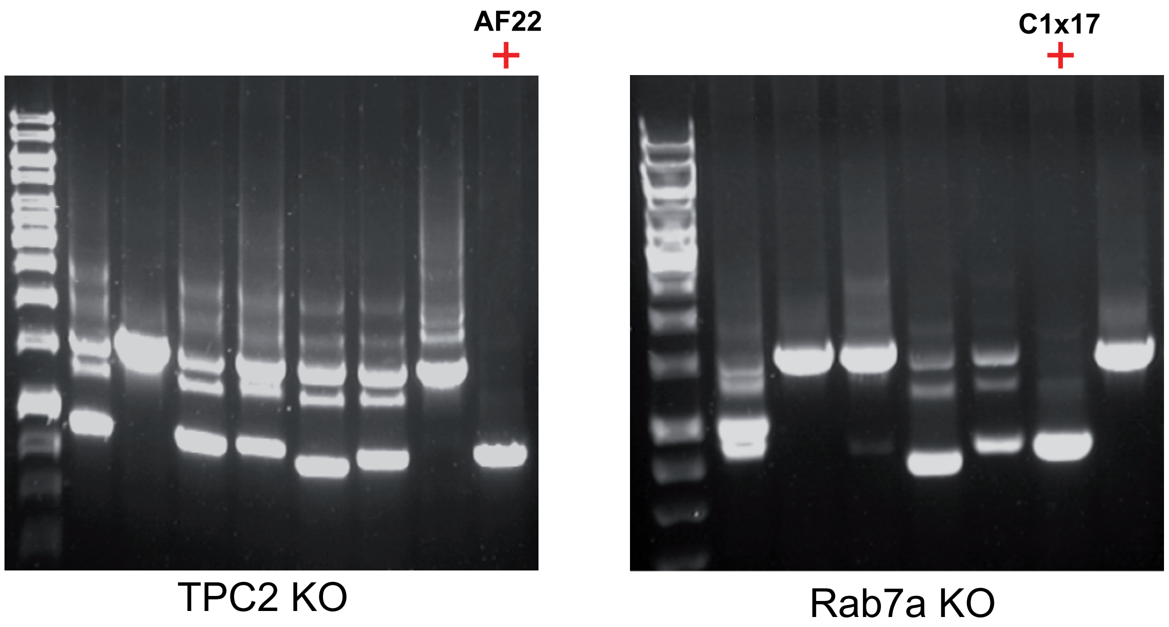

Genotyping (SK-MEL-5)

Figure S4. Genotyping results for Rab7a and TPC2 KO SK-MEL-5 cell lines. Agarose gels showing KO clone selection. Potential clones were sequenced. For all clones, homogeneity within the deletion amplicons was determined with Sanger sequencing and the CRISPR-ID *in silico* tool as described by Dehairs et al., 2016[2]. Genotyping results for the other two TPC2KO clones have been reported previously in Yuan et al., 2022[3].

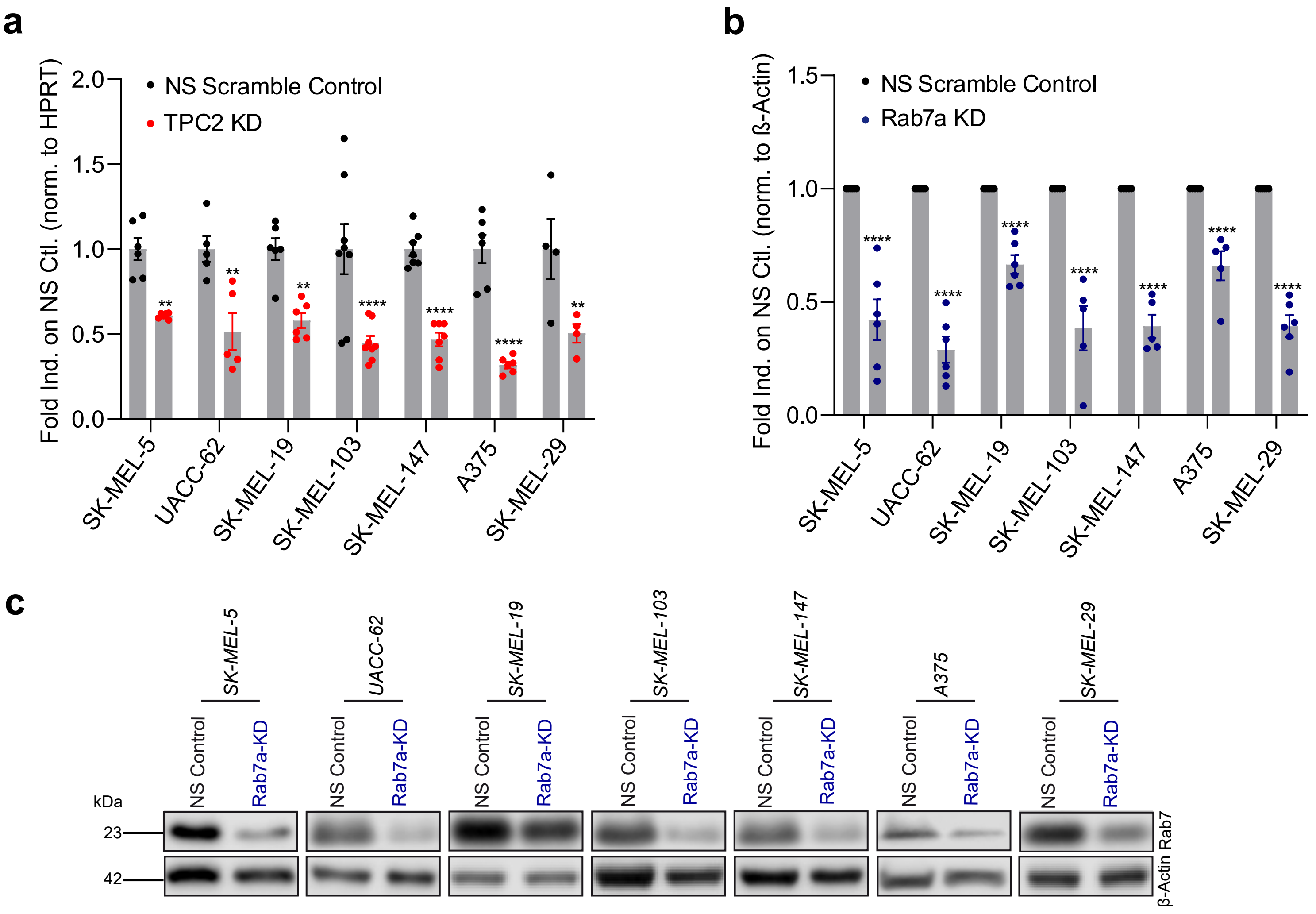
a
Fold Ind. on NS Ctl. (norm. to HPRT)
2.0
1.5
1.0
0.5
0.0
NS Scramble Control
TPC2 KD
**
**
**
****
****
****
**
SK-MEL-5
UACC-62
SK-MEL-19
SK-MEL-103
SK-MEL-147
A375
SK-MEL-29
b
Fold Ind. on NS Ctl. (norm. to ß-Actin)
1.5
1.0
0.5
0.0
NS Scramble Control
Rab7a KD
****
****
****
****
****
****
****
SK-MEL-5
UACC-62
SK-MEL-19
SK-MEL-103
SK-MEL-147
A375
SK-MEL-29
c
SK-MEL-5
UACC-62
SK-MEL-19
SK-MEL-103
SK-MEL-147
A375
SK-MEL-29
NS Control
Rab7a-KD
kDa
23
42
Rab7
ß-Actin

Figure S5. Knockdown efficiencies for different melanoma lines. (**a**) TPC2 KD in different melanoma lines determined by transcript levels using qPCR, fold induction on NS control, normalized to the house-keeping gene HPRT (n = 4-8 biological replicates). (**b**) Rab7 KD efficiency determined by Western blot experiments showing reduced proteins levels, fold induction on NS control, normalized to β-Actin (n = 5-6 biological replicates). (**c**) Representative blots for Rab7 KD experiments as shown in b. Statistical significance in a and b was determined using one-way ANOVA followed by Bonferroni multiple comparisons test. Shown are mean values ± SEM. $^{**}p < 0.01$, $^{****}p < 0.0001$.

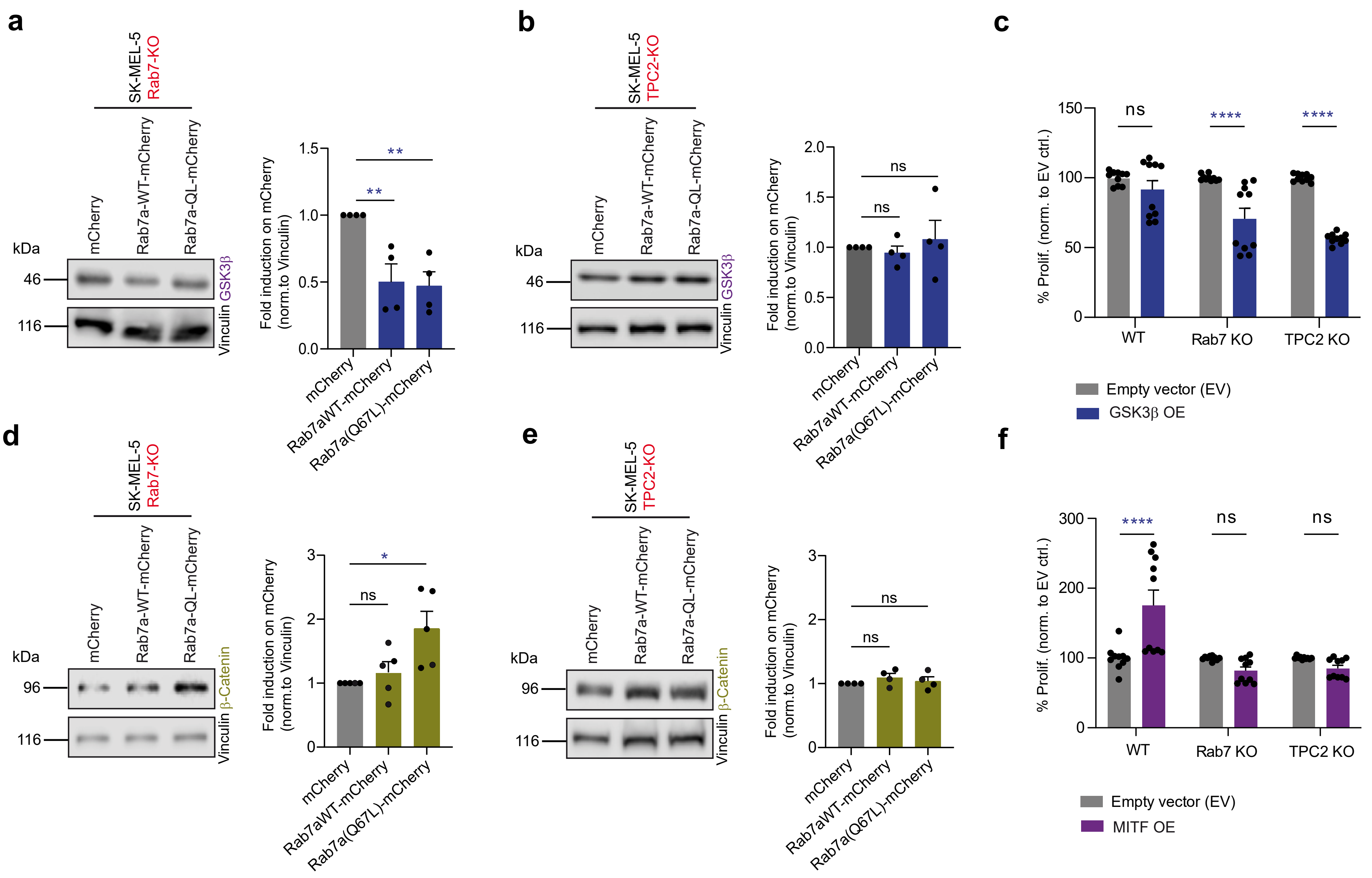

a
SK-MEL-5 Rab7-KO
mCherry
Rab7a-WT-mCherry
Rab7a-QL-mCherry
kDa
46
116
GSK3β
Vinculin
Fold induction on mCherry (norm.to Vinculin)
Rab7aWT-mCherry
Rab7a(Q67L)-mCherry
b
SK-MEL-5 TPC2-KO
ns
c
% Prolif. (norm. to EV ctrl.)
WT
Rab7 KO
TPC2 KO
Empty vector (EV)
GSK3β OE
d
β-Catenin
96
e
f
MITF OE

Figure S6. Effect of Rab7a OE on GSK3β expression in Rab7a and TPC2 KO and effect of GSK3β overexpression on Rab7a and TPC2 KO cell proliferation. (**a**) Overexpression of Rab7a-WT-mCherry and Rab7a-QL-mCherry results in decreased GSK3β protein levels in Rab7a KO SK-MEL-5 cells, respectively (n = 4 biological replicates). (**b**) Overexpression of Rab7a-WT-mCherry and Rab7a-QL-mCherry results in unchanged GSK3β protein levels in TPC2 KO SK-MEL-5 cells, respectively (n = 4 biological replicates). (**c**) Effect of overexpression of GSK3β in either WT, Rab7a or TPC2 KO SK-MEL-5 cells on proliferation (n = 10 biological replicates). (**d**) Overexpression of Rab7a-WT-mCherry and Rab7a-QL-mCherry results in increased β-Catenin protein levels in Rab7a KO SK-MEL-5 cells, respectively (n = 5 biological replicates). (**e**) Overexpression of Rab7a-WT-mCherry and Rab7a-QL-mCherry results in unchanged β-Catenin protein levels in TPC2 KO SK-MEL-5 cells, respectively (n = 4 biological replicates). (**f**) Effect of overexpression of MITF in either WT, Rab7a or TPC2 KO SK-MEL-5 cells on proliferation (n = 10 biological replicates). Statistical significance in a-f was determined using one-way ANOVA followed by Bonferroni multiple comparisons test. Shown are mean values ± SEM, *p < 0.05, **p < 0.01, ****p < 0.0001.

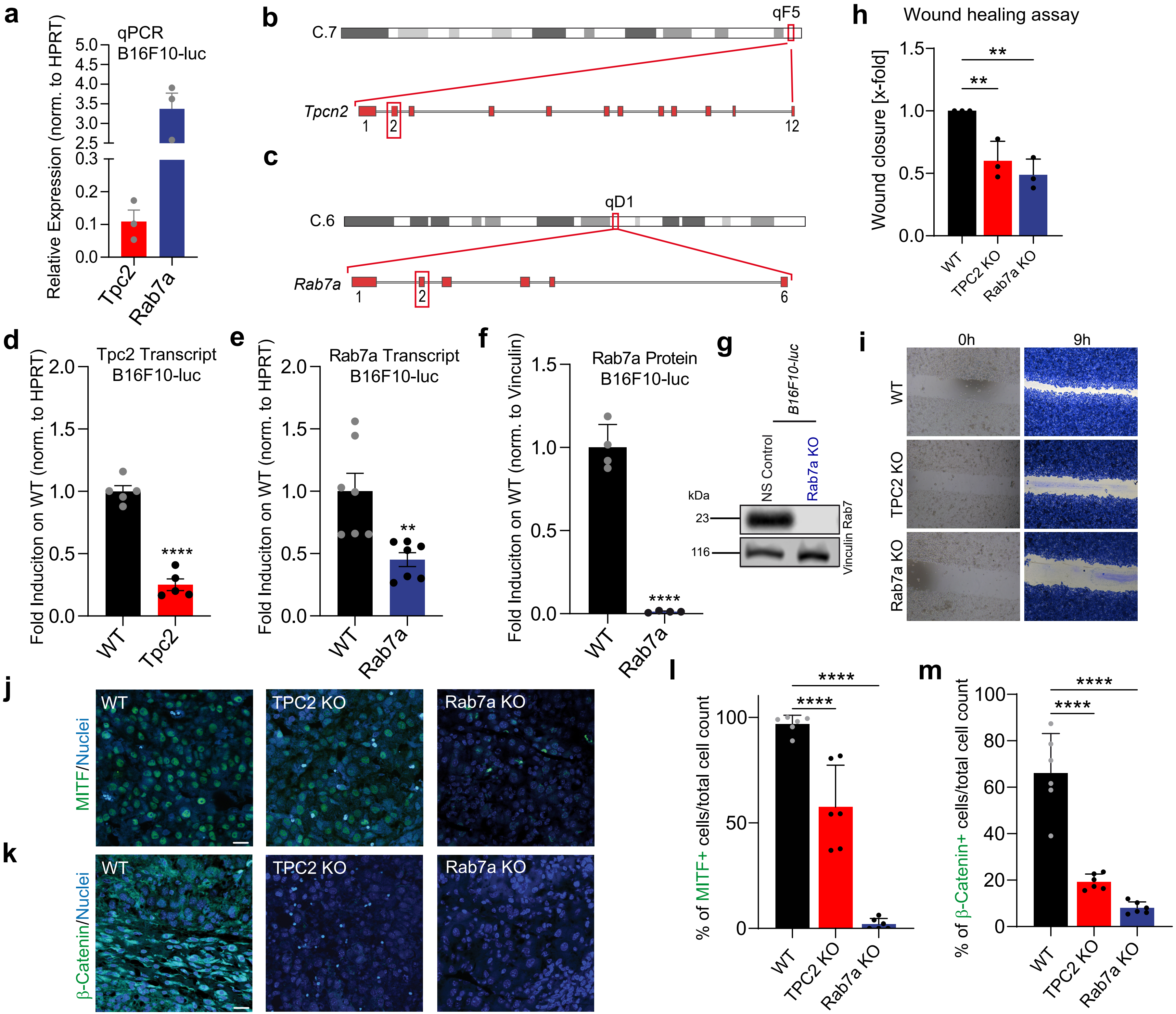
a
qPCR
B16F10-luc
Relative Expression (norm. to HPRT)
Tpc2
Rab7a
b
C.7
qF5
Tpcn2
c
C.6
qD1
Rab7a
h
Wound healing assay
Wound closure [x-fold]
WT
TPC2 KO
Rab7a KO
d
Tpc2 Transcript
B16F10-luc
Fold Induciton on WT (norm. to HPRT)
WT
Tpc2
e
Rab7a Transcript
B16F10-luc
Fold Induciton on WT (norm. to HPRT)
WT
Rab7a
f
Rab7a Protein
B16F10-luc
Fold Induciton on WT (norm. to Vinculin)
WT
Rab7a
g
B16F10-luc
NS Control
Rab7a KO
kDa
23
116
Rab7
Vinculin
i
0h
9h
WT
TPC2 KO
Rab7a KO
j
MITF/Nuclei
WT
TPC2 KO
Rab7a KO
k
β-Catenin/Nuclei
WT
TPC2 KO
Rab7a KO
l
% of MITF+ cells/total cell count
WT
TPC2 KO
Rab7a KO
m
% of β-Catenin+ cells/total cell count
WT
TPC2 KO
Rab7a KO

Figure S7. Characterization of B16F10-luc Tpc2 and Rab7a knockout lines. (**a**) Gene expression profile of TPC2 and Rab7a in the B16F10-luc cell line (n = 3 biological replicates). (**b** and **c**) CRISPR/Cas9 gene editing strategy used for the knockout of Tpcn2 (b) and Rab7 (c) in the B16F10-luc cells. (**d**) qPCR experiments showing reduced Tpcn2 transcript levels in the Tpc2 KO B16F10-luc cell line (n = 4-5 biological replicates). (**e**) qPCR experiments showing reduced Rab7a transcript levels in the Rab7a KO B16F10-luc cell line (n = 7 biological replicates). Statistical significance in d-e was determined using a two-tailed unpaired t-test. Shown are mean values ± SEM. **p < 0.01, ****p < 0.0001. (**f-g**) Western blot results for the Rab7a KO in B16F10-luc cells (n = 4 biological replicates). (**h-i**) Wound healing assay in B16F10-luc WT, Tpc2 KO and Rab7a KO showing reduced migration in Kos (n = 3 biological replicates). Statistical significance in f was determined using one-way ANOVA. Shown are mean values ± SD. **p < 0.01. (**j-k**) Confocal images of *ex vivo* B16F10-luc tumors. Tumours were fixed in paraffin, cut into 10 µm thin slices and stained for MITF or β-Catenin (green) and nuclei (blue), respectively. (**l-m**) Quantitative analysis of confocal images shown in j-k. Data were obtained from 3 different tumors (duplicates), each. In total 10,676 (WT), 7675 (TPC2 KO) and 4299 (Rab7 KO) cells were analyzed for MITF and 3946 (WT), 4022 (TPC2 KO) and 4793 (Rab7 KO) cells were analyzed for β-Catenin. Statistical significance in l-m was assessed with one-way ANOVA followed by Bonferroni multiple comparisons test. Shown are mean values ± SEM, ****p < 0.0001. Scale bar = 100 µm.

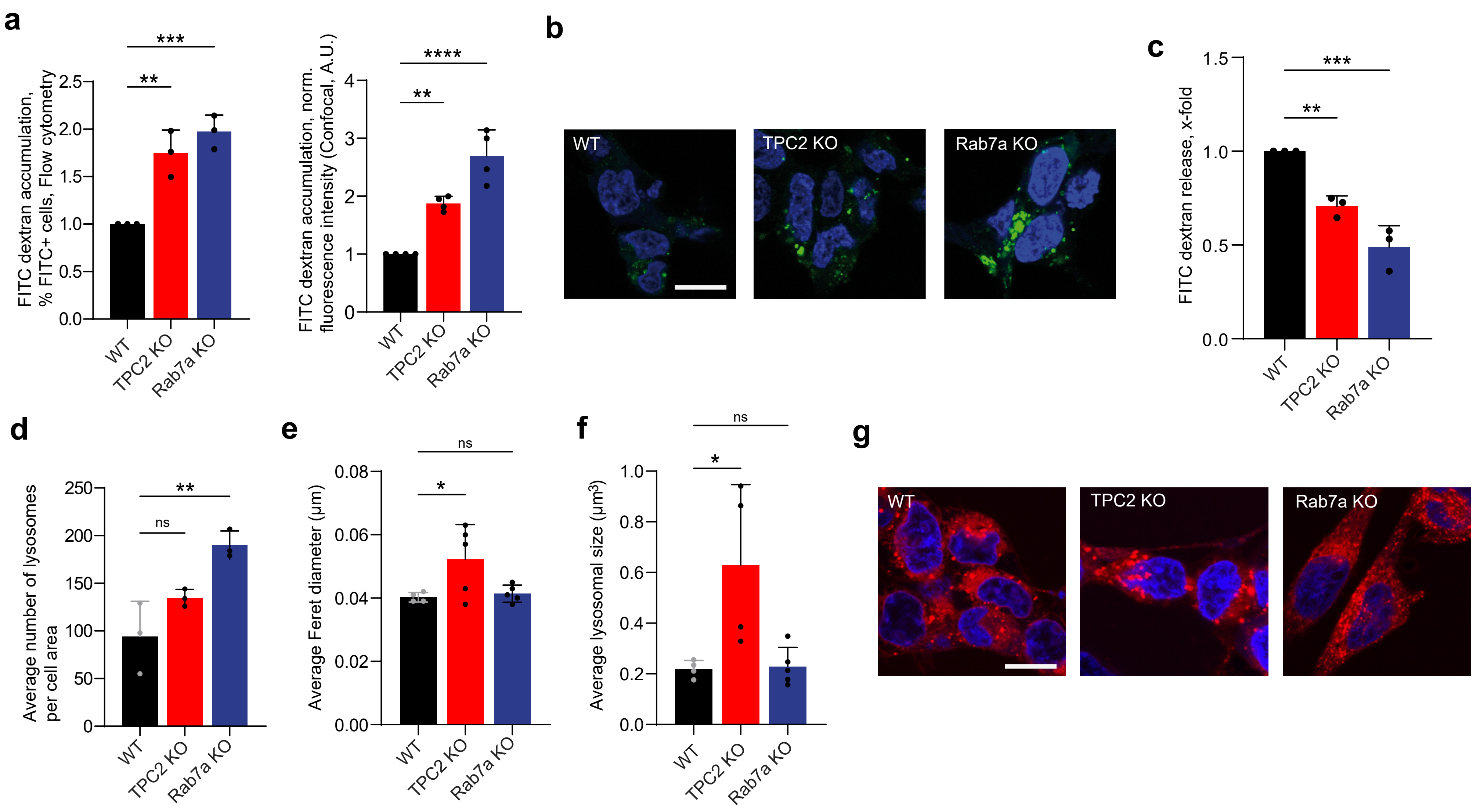
a
FITC dextran accumulation, % FITC+ cells, Flow cytometry
FITC dextran accumulation, norm. fluorescence intensity (Confocal, A.U.)
WT
TPC2 KO
Rab7a KO
b
WT
TPC2 KO
Rab7a KO
c
FITC dextran release, x-fold
d
Average number of lysosomes per cell area
ns
e
Average Feret diameter (µm)
f
Average lysosomal size (µm³)
g
WT
TPC2 KO
Rab7a KO

Figure S8. Lysosomal defects in Rab7a and TPC2 KO SK-MEL-5 cell lines. (**a**) Flow cytometry and confocal analysis of endocytosed FITC dextran (200 µg/ml) after an incubation time of 2 h (n = 3-4 biological replicates). (**b**) Confocal images showing internalized FITC dextran (200 µg/ml, incubated for 2 h) and nuclei. Fluorescence intensities were measured by ImageJ and normalized to the number of cells per image. Scale bar = 10µm. (**c**) Lysosomal exocytosis assay of released FITC-dextran (200 µg/mL, incubated for 24 h) upon calcium treatment (50 mM). The results were normalized to the WT level (n = 3 biological replicates). (**d**) Average amount of lysosomes per cell area (n = 3 biological replicates). (**e**) Average Feret diameters (n = 5 biological replicates). (**f**) Average size of lysosomes per cell. Data were pooled from four independent experiments (d-f). One dot represents one experiment. A total of 33189 (WT), 40905 (TPC2 KO) and 42414 (Rab7a KO) lysosomes were evaluated. (**g**) Representative images of WT, TPC2 KO and Rab7a KO SK-MEL-5 cells treated with Lysotracker Red and Hoechst (nuclei). Statistical significance in a, c, d, e, f was assessed by one-way ANOVA followed by Bonferroni multiple comparisons test. Shown are mean values ± SEM. $p < 0.05$, **$p < 0.01$, ***$p < 0.001$.****$p < 0.0001$. Scale bar = 10 µm.

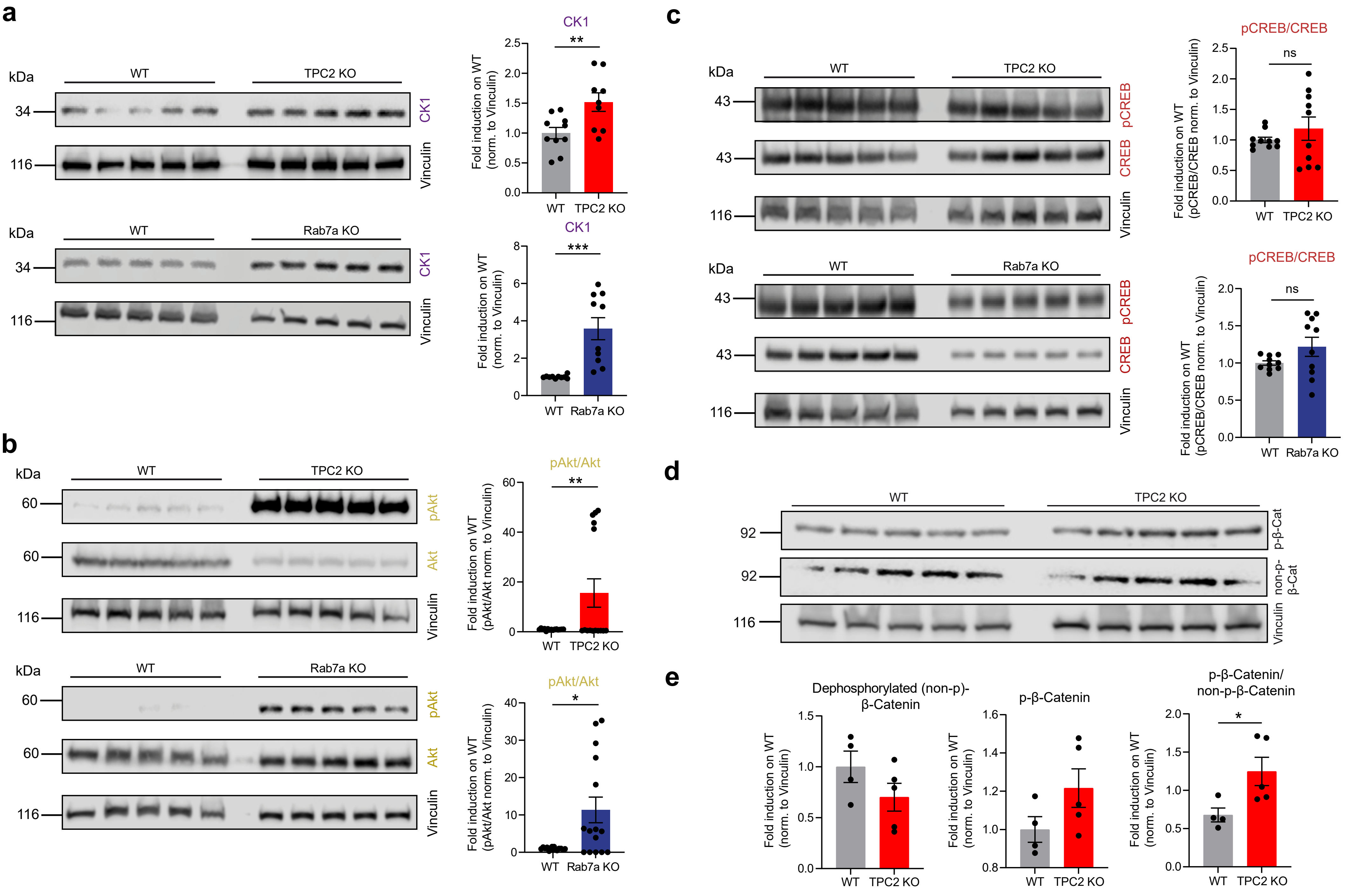

a
kDa
WT
TPC2 KO
34
116
CK1
Vinculin
CK1
**
Fold induction on WT (norm. to Vinculin)
0.0
0.5
1.0
1.5
2.0
2.5
WT
TPC2 KO
kDa
WT
Rab7a KO
34
116
CK1
Vinculin
CK1
***
Fold induction on WT (norm. to Vinculin)
0
2
4
6
8
WT
Rab7a KO
b
kDa
WT
TPC2 KO
60
60
116
pAkt
Akt
Vinculin
pAkt/Akt
**
Fold induction on WT (pAkt/Akt norm. to Vinculin)
0
20
40
60
WT
TPC2 KO
kDa
WT
Rab7a KO
60
60
116
pAkt
Akt
Vinculin
pAkt/Akt
*
Fold induction on WT (pAkt/Akt norm. to Vinculin)
0
10
20
30
40
WT
Rab7a KO
c
kDa
WT
TPC2 KO
43
43
116
pCREB
CREB
Vinculin
pCREB/CREB
ns
Fold induction on WT (pCREB/CREB norm. to Vinculin)
0.0
0.5
1.0
1.5
2.0
2.5
WT
TPC2 KO
kDa
WT
Rab7a KO
43
43
116
pCREB
CREB
Vinculin
pCREB/CREB
ns
Fold induction on WT (pCREB/CREB norm. to Vinculin)
0.0
0.5
1.0
1.5
2.0
WT
Rab7a KO
d
WT
TPC2 KO
92
92
116
p-β-Cat
non-p-β-Cat
Vinculin
e
Dephosphorylated (non-p)-β-Catenin
Fold induction on WT (norm. to Vinculin)
0.0
0.5
1.0
1.5
WT
TPC2 KO
p-β-Catenin
Fold induction on WT (norm. to Vinculin)
0.8
1.0
1.2
1.4
1.6
WT
TPC2 KO
p-β-Catenin/ non-p-β-Catenin
*
Fold induction on WT (norm. to Vinculin)
0.0
0.5
1.0
1.5
2.0
WT
TPC2 KO

Figure S9. Effect of Rab7a and TPC2 KO on CK1, pCREB/CREB and pAkt/Akt. (**a-c**) Representative Western blots for CK1 (n = 9-10 biological replicates), pCREB/CREB (n = 15 biological replicates) and pAkt/Akt (n = 10 biological replicates) protein expression in SK-MEL-5 WT, Rab7a KO and TPC2 KO lines and respective statistical analysis: significance was determined using a two-tailed unpaired t-test. Shown are mean values ± SEM. *$p < 0.05$, **$p < 0.01$, ***$p < 0.001$. (**d-e**) Representative Western blots for dephosphorylated and phospho-β-Catenin in TPC2 KO compared to WT SK-MEL5 cells (n = 4-5 biological replicates). Significance was determined using a two-tailed unpaired t-test. Shown are mean values ± SEM. *$p < 0.05$.

1. Feldmann, C., et al., *Protocol for deriving proximity, affinity, and stoichiometry of protein interactions using image-based quantitative two-hybrid FRET.* STAR Protoc, 2023. **4**(3): p. 102459.
2. Dehairs, J., et al., *CRISP-ID: decoding CRISPR mediated indels by Sanger sequencing.* Sci Rep, 2016. **6**: p. 28973.
3. Yuan, Y., et al., *Segregated cation flux by TPC2 biases Ca(2+) signaling through lysosomes.* Nat Commun, 2022. **13**(1): p. 4481.

Figure S1e - myc
HEK293

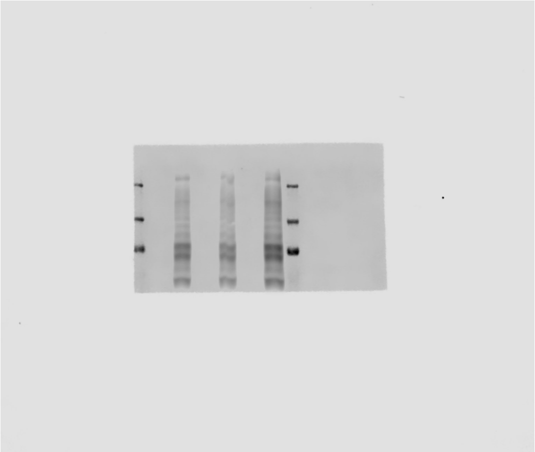

Figure S1e - mcherry
HEK293

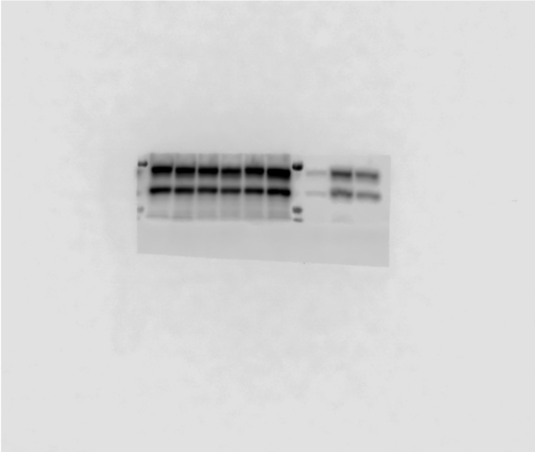

Figure S1e - myc
HEK293

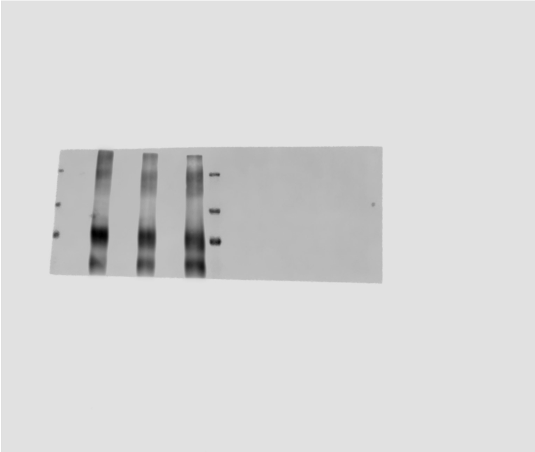

Figure S1e - myc
HEK293

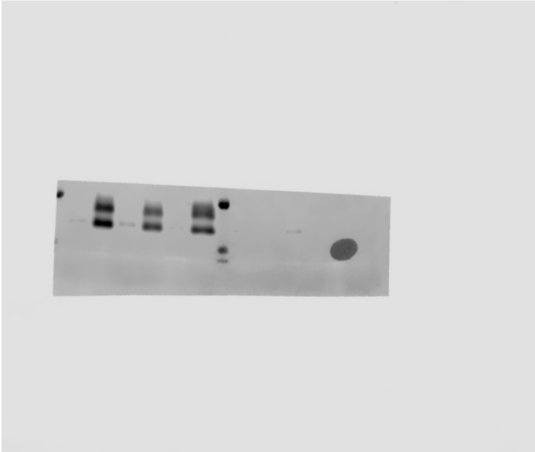

Figure S10. Unprocessed western blots of figure S1e. Co-immunoprecipitation experiment indicating an interaction between both hRab7aWT-mCherry and hTPC2WT-YFP and hRab7aQ67L-mCherry with hTPC2WT-YFP1.

Figure S5c - Rab7

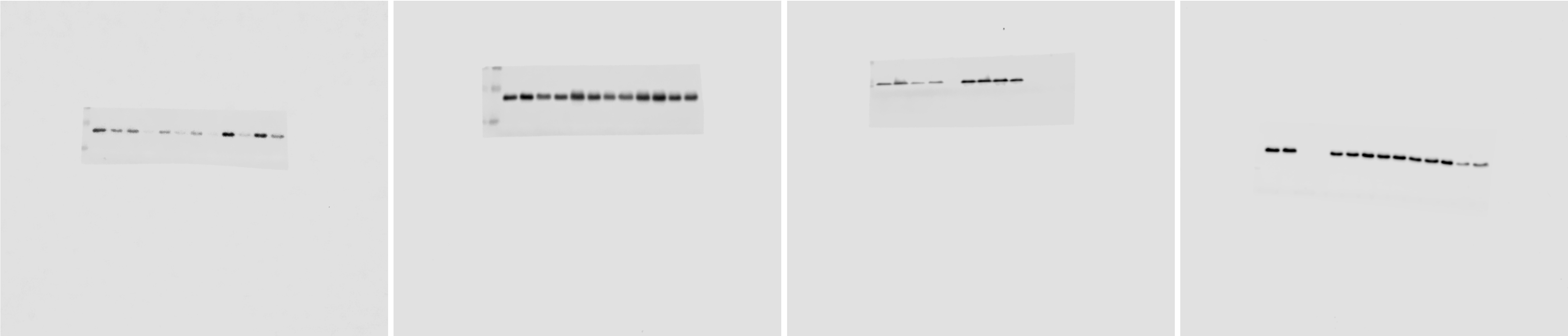

Figure S5c - Β-Actin

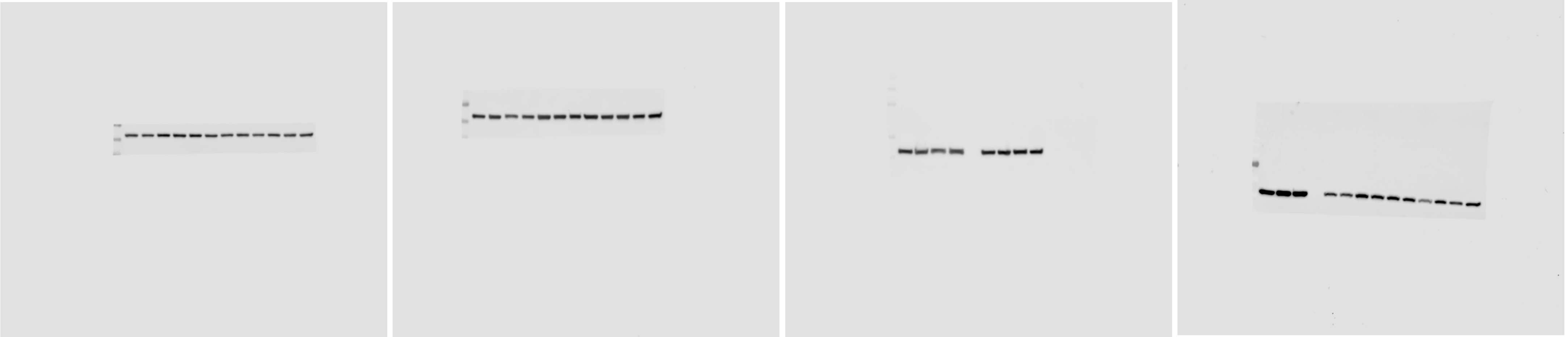

Figure S6a - Vinculin

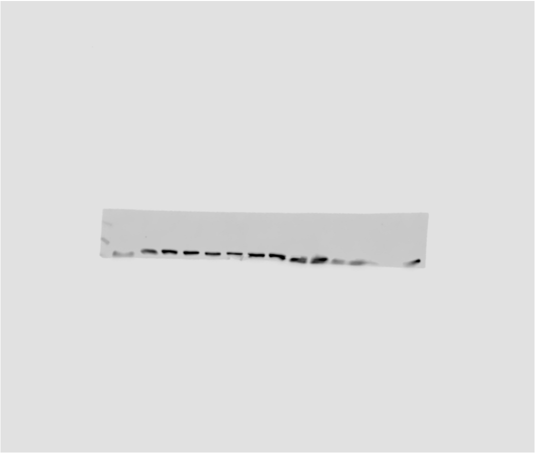

Figure S6a - GSK3b

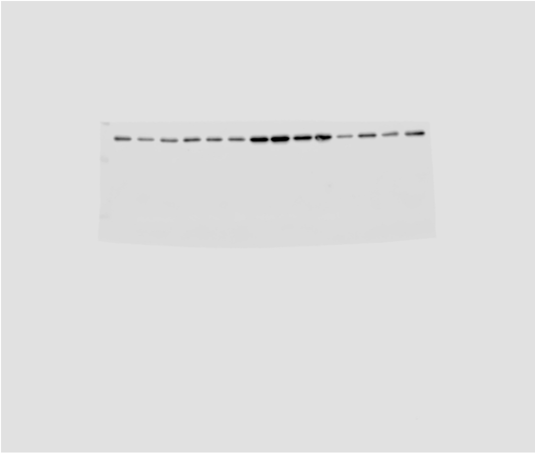

Figure S6b - Vinculin

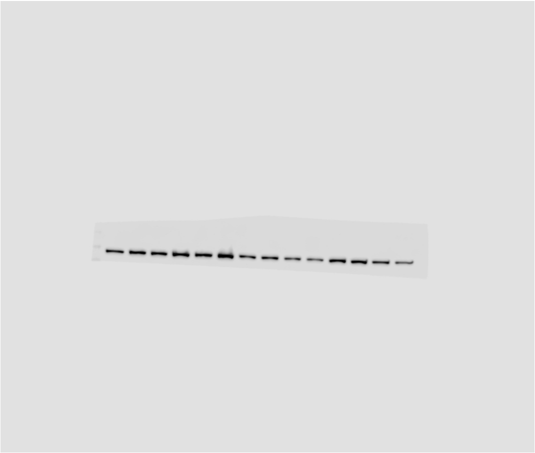

Figure S6b - GSK3b

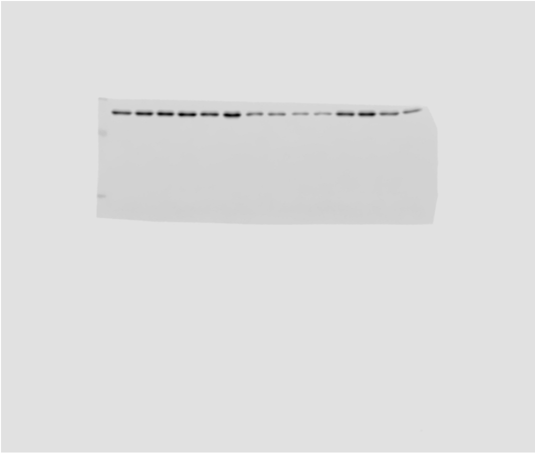

Figure S11. Unprocessed western blots of figures S5c, S6a and S6b. Representative blots for Rab7 KD experiments (S5c). Overexpression of Rab7a-WT-mCherry and Rab7a-QL-mCherry results in decreased GSK3β protein levels in Rab7a KO SK-MEL-5 cells, respectively (S6a). Overexpression of Rab7a-WT-mCherry and Rab7a-QL-mCherry results in unchanged GSK3β protein levels in TPC2 KO SK-MEL-5 cells, respectively (S6b).

Figure S6d - beta-Catenin

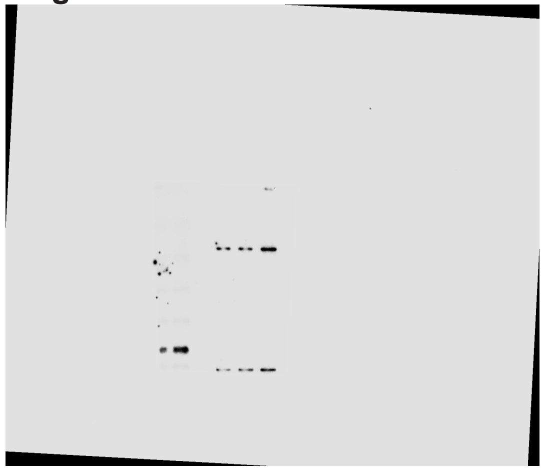

Figure S6d - Vinculin

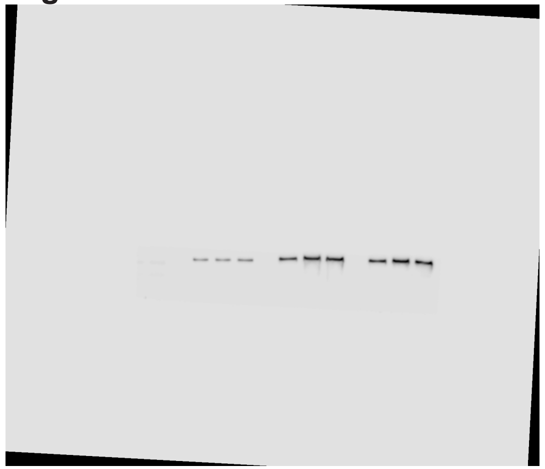

Figure S6e - beta-catenin

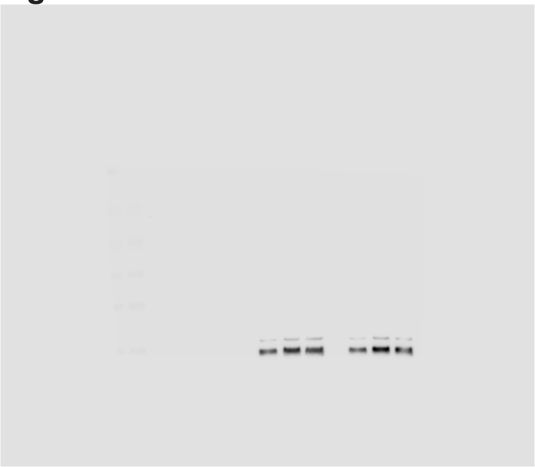

Figure S6e - Vinculin

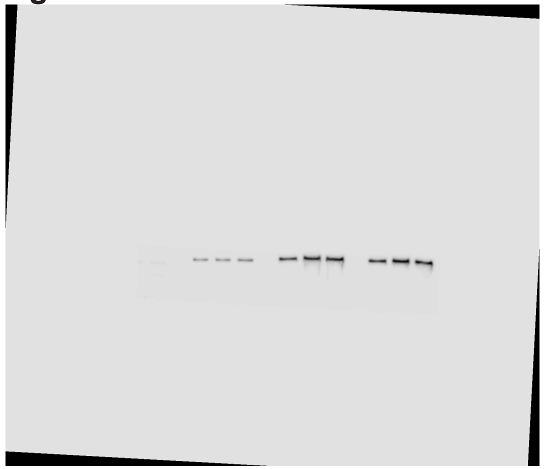

Figure S12. Unprocessed western blots of figures S6d and S6e. Overexpression of Rab7a-WT-mCherry and Rab7a-QL-mCherry results in increased β-Catenin protein levels in Rab7a KO SK-MEL-5 cells, respectively (S6d). Overexpression of Rab7a-WT-mCherry and Rab7a-QL-mCherry results in unchanged β-Catenin protein levels in TPC2 KO SK-MEL-5 cells, respectively (S6e).

Figure S7g - Rab7

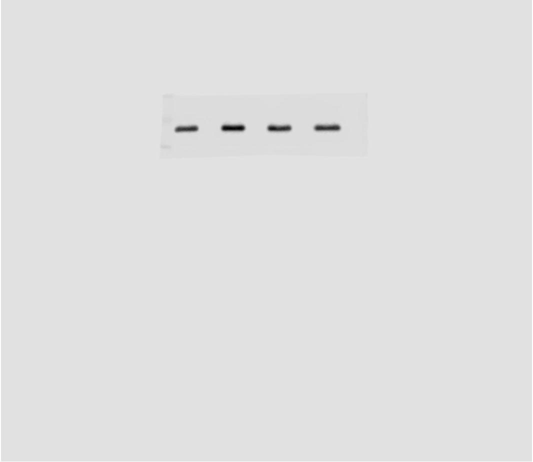

Figure S7g - Vinculin

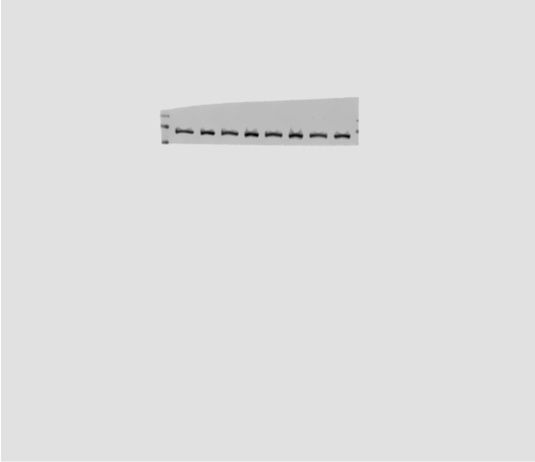

Figure S9a - CK1, TPC2 KO

Figure S9a - Vinculin, TPC2 KO

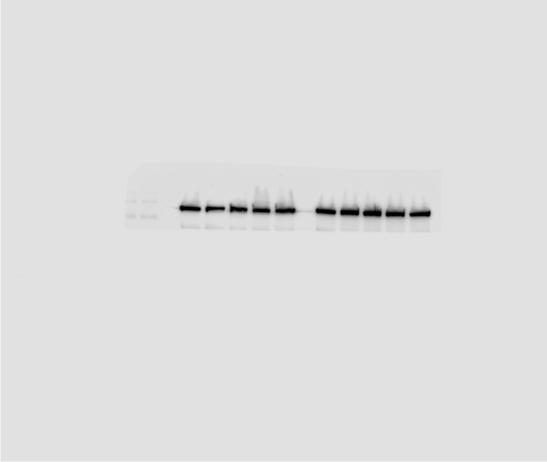

Figure S9a - CK1, Rab7 KO

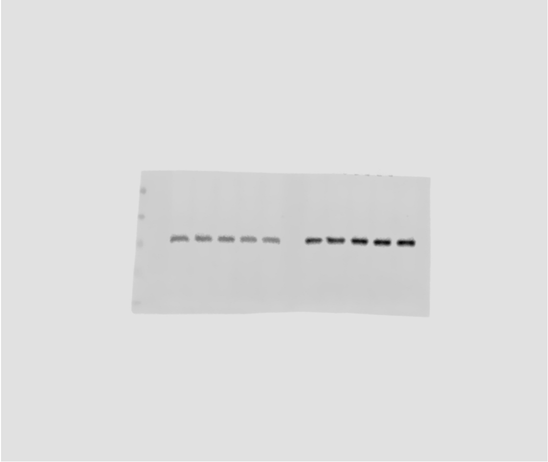

Figure S9a - Vinculin, Rab7 KO

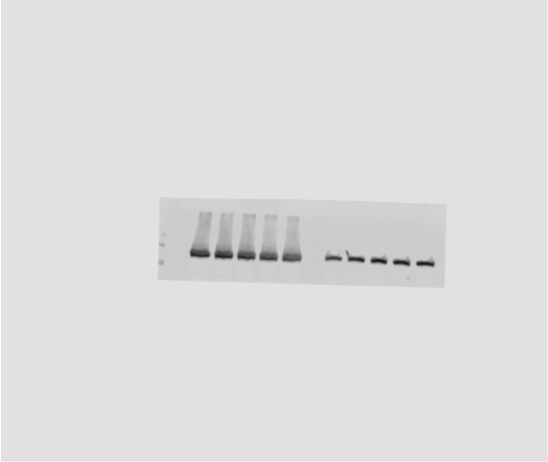

Figure S9b - p-Akt, TPC2 KO

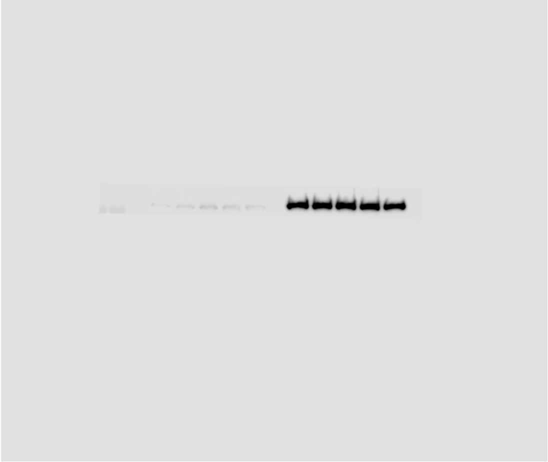

Figure S9b - Akt, TPC2 KO

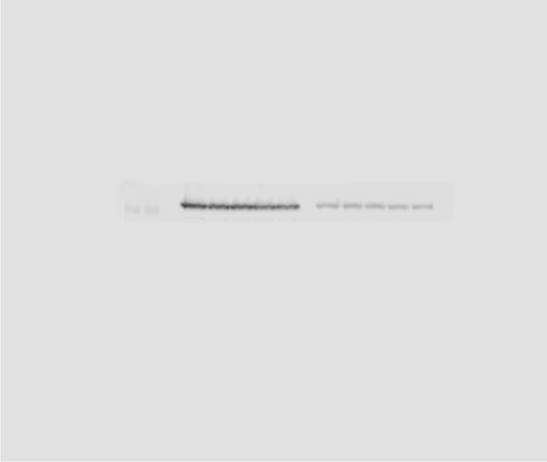

Figure S9b - Vinculin TPC2 KO

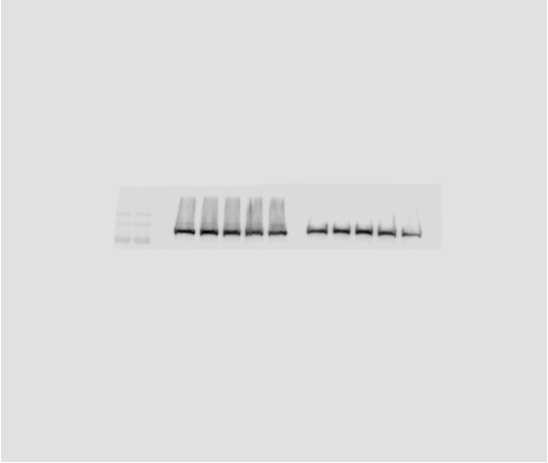

Figure S9b - p-Akt, Rab7 KO

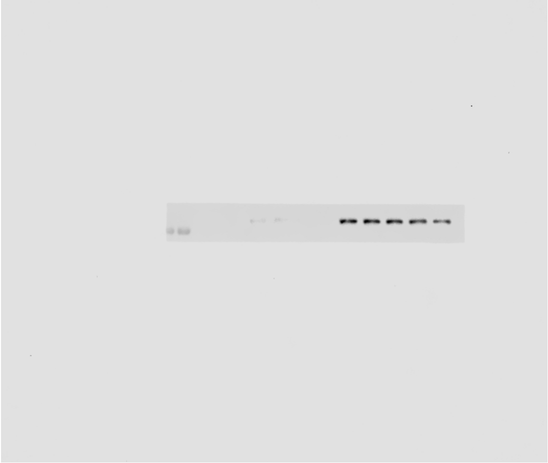

Figure S9b - Akt, Rab7 KO

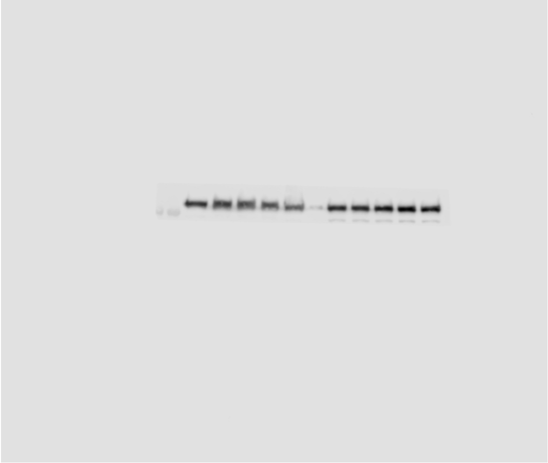

Figure S9b - Vinculin, Rab7 KO

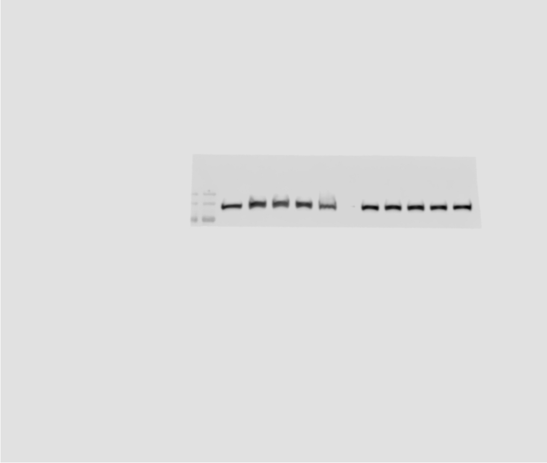

Figure S13. Unprocessed western blots of figures S7g, S9a and S9b. Western blot results for the Rab7a KO in B16F10-luc cells (S7g). Western blots for CK1 (S9a) and pAkt/Akt (S9b) protein expression in SK-MEL-5 WT, Rab7a KO and TPC2 KO lines.

Figure S9c - p-CREB, TPC2 KO

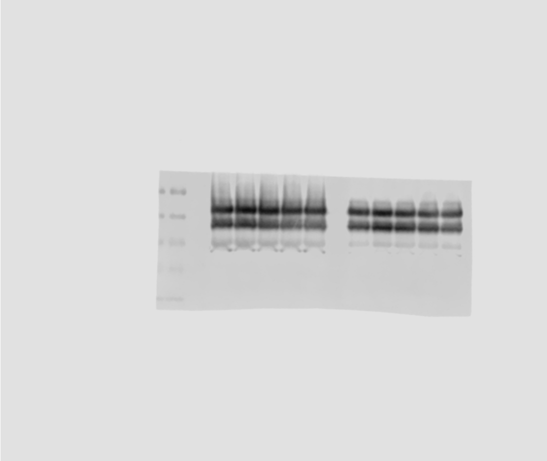

Figure S9c - CREB, TPC2 KO

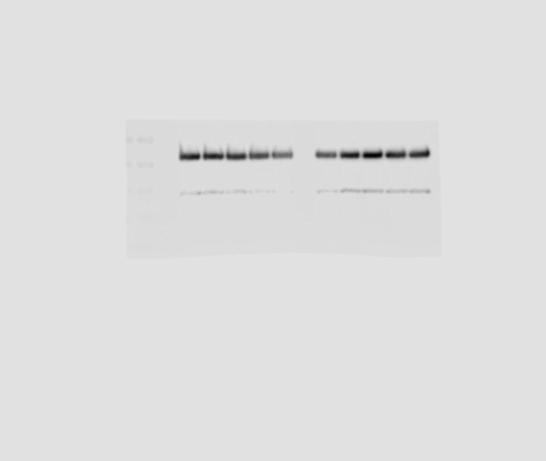

Figure S9c - Vinculin, TPC2 KO

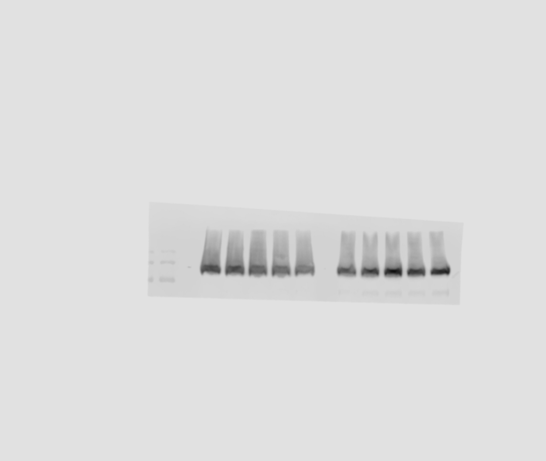

Figure S9c - p-beta-Catenin

Figure S9c - p-CREB, Rab7 KO

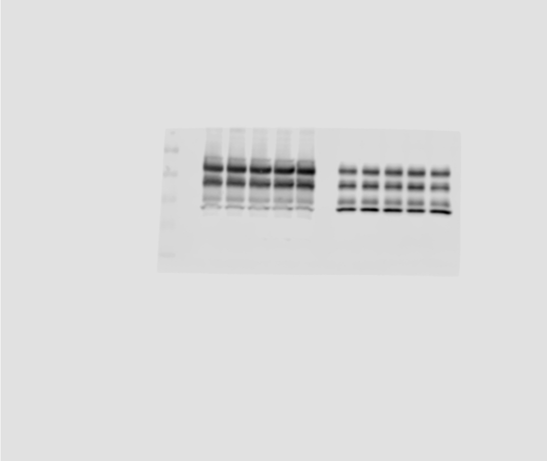

Figure S9c - CREB, Rab7 KO

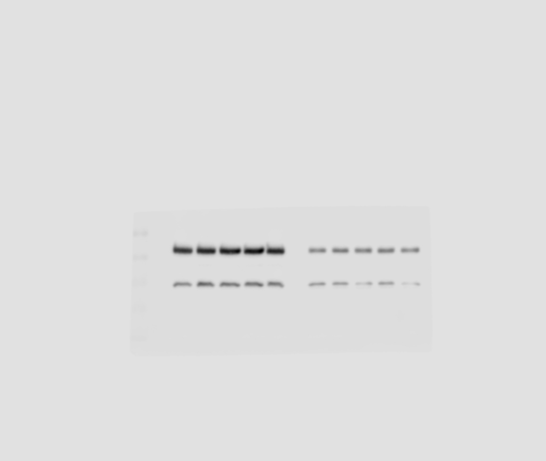

Figure S9c - Vinculin, Rab7 KO

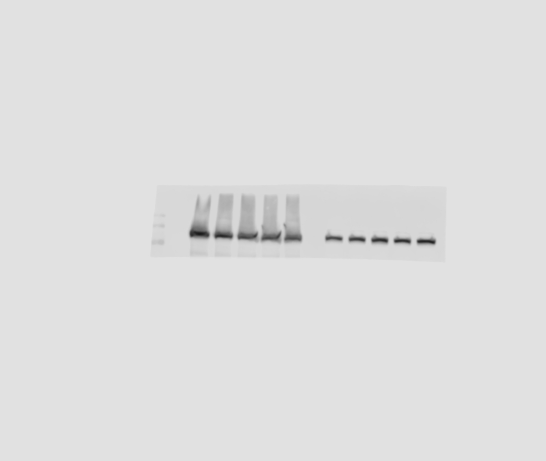

Figure S9d - non-p-beta-Catenin

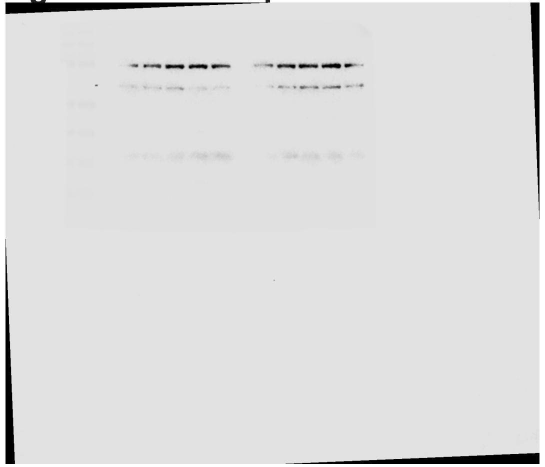

Figure S9d - Vinculin

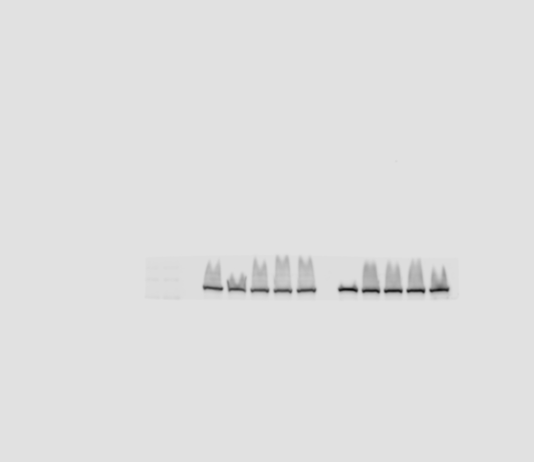

Figure S14. Unprocessed western blots of figures S9c and S9d. Western blots for pCREB/CREB protein expression in SK-MEL-5 WT, Rab7a KO and TPC2 KO lines (S9c). Western blots for dephosphorylated and phospho-β-Catenin in TPC2 KO compared to WT SK-MEL5 cells (S9d).
