# Supplementary material for: Rab7a is an enhancer of TPC2 activity regulating melanoma progression through modulation of the GSK3β/β-Catenin/MITF-axis
Source: Nat Commun. 2024 Nov 19;15:10008. doi: 10.1038/s41467-024-54324-9 (PMC11576762; doi:10.1038/s41467-024-54324-9)
Supplement: Supplementary file 2 — Description of Additional Supplementary Files [file 41467_2024_54324_MOESM2_ESM.pdf]

Description of Additional Supplementary Files

Supplementary Data 1. Antibodies, Oligonucleotides, Cell lines
