# Supplementary material for: Rab7a is an enhancer of TPC2 activity regulating melanoma progression through modulation of the GSK3β/β-Catenin/MITF-axis
Source: Nat Commun. 2024 Nov 19;15:10008. doi: 10.1038/s41467-024-54324-9 (PMC11576762; doi:10.1038/s41467-024-54324-9)
Supplement: Supplementary file 4 — Reporting Summary [file 41467_2024_54324_MOESM4_ESM.pdf]

nature portfolio

Corresponding author(s): Christian Grimm, Karin Bartel

Last updated by author(s): Sep 13, 2024

Reporting Summary

Nature Portfolio wishes to improve the reproducibility of the work that we publish. This form provides structure for consistency and transparency in reporting. For further information on Nature Portfolio policies, see our Editorial Policies and the Editorial Policy Checklist.

Statistics

For all statistical analyses, confirm that the following items are present in the figure legend, table legend, main text, or Methods section.

n/a	Confirmed	
☐	☒	The exact sample size (*n*) for each experimental group/condition, given as a discrete number and unit of measurement
☐	☒	A statement on whether measurements were taken from distinct samples or whether the same sample was measured repeatedly
☐	☒	The statistical test(s) used AND whether they are one- or two-sided<br>*Only common tests should be described solely by name; describe more complex techniques in the Methods section.*
☒	☐	A description of all covariates tested
☐	☒	A description of any assumptions or corrections, such as tests of normality and adjustment for multiple comparisons
☐	☒	A full description of the statistical parameters including central tendency (e.g. means) or other basic estimates (e.g. regression coefficient) AND variation (e.g. standard deviation) or associated estimates of uncertainty (e.g. confidence intervals)
☐	☒	For null hypothesis testing, the test statistic (e.g. *F*, *t*, *r*) with confidence intervals, effect sizes, degrees of freedom and *P* value noted<br>*Give P values as exact values whenever suitable.*
☒	☐	For Bayesian analysis, information on the choice of priors and Markov chain Monte Carlo settings
☒	☐	For hierarchical and complex designs, identification of the appropriate level for tests and full reporting of outcomes
☒	☐	Estimates of effect sizes (e.g. Cohen's *d*, Pearson's *r*), indicating how they were calculated

Our web collection on statistics for biologists contains articles on many of the points above.

Software and code

Policy information about availability of computer code

Data collection	- in vivo: Living Image 4.4 software (Perkin Elmer)<br>- Western blot: Image Lab 6.0.1 (Bio-Rad)<br>- QuPath-0.4.4 (console)<br>- qPCR: Thermo Fisher Connect
Data analysis	- scratch assay& confocal images: ImageJ software 1.53k (NIH)<br>- in vivo: Living Image 4.4 software (Perkin Elmer)<br>- Microsoft Excel 20 (version 1908 Microsoft)<br>- GraphPad Prism 10.0<br>- Western Blot: Image Lab 6.0.1 (Bio-Rad)<br>- QuPath-0.4.4 (console)<br><br>- qPCR: Thermo Fisher Connect<br>- Wound healing assay: ImageJ

For manuscripts utilizing custom algorithms or software that are central to the research but not yet described in published literature, software must be made available to editors and reviewers. We strongly encourage code deposition in a community repository (e.g. GitHub). See the Nature Portfolio guidelines for submitting code & software for further information.

Data

Policy information about availability of data

All manuscripts must include a data availability statement. This statement should provide the following information, where applicable:

- Accession codes, unique identifiers, or web links for publicly available datasets
- A description of any restrictions on data availability
- For clinical datasets or third party data, please ensure that the statement adheres to our policy

Raw data underlying the figures are available as excel sheets in the source data.

Research involving human participants, their data, or biological material

Policy information about studies with human participants or human data. See also policy information about sex, gender (identity/presentation), and sexual orientation and race, ethnicity and racism.

Reporting on sex and gender	We obtained human samples form archived samples of the department of pathology of the Ludwig Maximilians University Munich. The formalin-fixed samples were randomly selected by the department of pathology (50/50 m/f donors, age 48-88 years old). The experiments are in accordance with ethical standards of the responsible committee on human experimentation (written approval from the ethics committee of the Ludwig Maximilian University Hospital, Munich, number 23-0119) and with the Helsinki Declaration of 1975, as revised in 2000.
Reporting on race, ethnicity, or other socially relevant groupings	Race, ethnicity or socially relevent groups were not considered for the present study. An initial analysis demonstrated that there were no significant differences between male and female participants, therefore we pooled the results for a more cohesive understanding of the hypothesis. Also the sample size of male and female participants is relatively small. We obtained pre-archived patient material that has been obtained with patient consent previously in accordance with ethical standards. Please see below.
Population characteristics	10 metastatic lymph nodes from melanoma patients (m: n=6, w: n=4) and healthy lymph nodes (m: n=4, w: n=6)
Recruitment	not applicable
Ethics oversight	The experiments are in accordance with ethical standards of the responsible committee on human experimentation (written approval from the ethics committee of the Ludwig Maximilian University Hospital, Munich, number 23-0119) and with the Helsinki Declaration of 1975, as revised in 2000.

Note that full information on the approval of the study protocol must also be provided in the manuscript.

Field-specific reporting

Please select the one below that is the best fit for your research. If you are not sure, read the appropriate sections before making your selection.

☒ Life sciences ☐ Behavioural & social sciences ☐ Ecological, evolutionary & environmental sciences

For a reference copy of the document with all sections, see nature.com/documents/nr-reporting-summary-flat.pdf

Life sciences study design

All studies must disclose on these points even when the disclosure is negative.

Sample size	No statistical methods were used to predetermine sample size for in vitro experiments. Sample sizes were determined by magnitude and consistency of measurable differences. Samples sizes for in vitro and in vivo experiments are fully described in the manuscript. For in vivo experiments sample size was determined by the institute of statistics of the LMU Munich according to Ackermann et al. 2006. Sample size was set to N=17 mice per group for tumor growth experiments and N=10 for tumor dissemination experiments. For in vitro experiments, at least triplicates were used.
Data exclusions	- in vivo experiments: ectopic tumor model (Bioluminescence imaging): ROUT outlier test [Q=1%] was performed to assess outliers in our cohort. The exclusion criteria was pre-established.
Replication	All experiments were repeated at least three times (biological triplicate), except for in vivo experiments that were performed once with the pre-defined group size of at least 17 or 10 mice per group respecitvely. All attempts at replication were sucessful.
Randomization	for all in vivo experiments, mice were randomized prior to engraftment of tumor cells or treatment. To controll covariates all mice were purchased from the same vendor, have the same age , bread and sex. Moreover, experiments were taken out at the same time, to minimize covariates.
Blinding	Investigators were not blinded during data collection and/or analysis as blinding was not relevant to this study. All values were determined by methods independent of operator bias.

Reporting for specific materials, systems and methods

We require information from authors about some types of materials, experimental systems and methods used in many studies. Here, indicate whether each material, system or method listed is relevant to your study. If you are not sure if a list item applies to your research, read the appropriate section before selecting a response.

Materials & experimental systems

n/a	Involved in the study	
☐	☒	Antibodies
☐	☒	Eukaryotic cell lines
☒	☐	Palaeontology and archaeology
☐	☒	Animals and other organisms
☒	☐	Clinical data
☒	☐	Dual use research of concern
☒	☐	Plants

Methods

n/a	Involved in the study	
☒	☐	ChIP-seq
☒	☐	Flow cytometry
☒	☐	MRI-based neuroimaging

Antibodies

Antibodies used	IHC: anti-MITF (Cell Signaling Technology, #12590, diluted 1:150) and anti-β-Catenin (Cell Signaling Technology, #8480, diluted 1:150) Human tissue samples: 1:160 (Rab7, Cell Signaling, #95746) or 1:100 (MITF, Agilent, M3621)
Validation	all antibodies are commercially available, widely used

Eukaryotic cell lines

Policy information about cell lines and Sex and Gender in Research

Cell line source(s)	B16F10luc cells were purchased at ATCC (CRL-6475-LUC2).
Authentication	Cell Line authentification was performed by STR profiling
Mycoplasma contamination	All cells are proven to be mycoplasma-free on a quarterly basis.
Commonly misidentified lines (See ICLAC register)	no commonly misidentified cell lines were used for this study

Animals and other research organisms

Policy information about studies involving animals; ARRIVE guidelines recommended for reporting animal research, and Sex and Gender in Research

Laboratory animals	- Dissemination assay: 30 C57Bl/6-Tyr mice (Envigo), female, 5-6 weeks old<br>- Ectoptic tumor model: 119 C57Bl/6-Tyr mice (Envigo), female, 5-6 weeks old
Wild animals	not applicable
Reporting on sex	The animal models in this study are based on female C57BL/6BrdCrHsd-Tyrc cancer models established in our lab (Müller et al, DOI: 10.1016/j.chembiol.2021.01.023; Ardelt et al. DOI: 10.1002/hep.30190). The present study aimed to investigate the pharmacological influence of Rab7a on TPC2 and thereby represents a mechanistic, preclinical study. The investigation of influence of sex or gender on the interaction between these proteins is beyond the scope of this publication. Additionally, to investigate sex influence on an interaction, the interaction itself must be proven beforehand, which was the focus of this publication. Of note, as the interaction itself was unknown thus far, ethical approval for mouse numbers to include sex analysis in the study would not have been provided by the district government of upper Bavaria as it contradicts the 3R principle.
Field-collected samples	not applicable
Ethics oversight	All animal experiments were approved by the District Government of Upper Bavaria, in accordance with institutional guidelines and the German Animal Welfare. Approval number ROB-55.2-2532.Vet_02-22-5

Note that full information on the approval of the study protocol must also be provided in the manuscript.

Plants

Seed stocks	not applicable
Novel plant genotypes	not applicable
Authentication	not applicable
